# Occurrence, molecular characterization and predominant genotypes of *Enterocytozoon bieneusi* in dairy cattle in Henan and Ningxia, China

**DOI:** 10.1186/s13071-016-1425-5

**Published:** 2016-03-11

**Authors:** Junqiang Li, Nannan Luo, Chenrong Wang, Meng Qi, Jianke Cao, Zhaohui Cui, Jianying Huang, Rongjun Wang, Longxian Zhang

**Affiliations:** College of Animal Science and Veterinary Medicine, Henan Agricultural University, Zhengzhou, 450002 China; International Joint Research Laboratory for Zoonotic Diseases of Henan, Zhengzhou, 450002 China

**Keywords:** *Enterocytozoon bieneusi*, Dairy cattle, ITS genotypes

## Abstract

**Background:**

*Enterocytozoon bieneusi* is the most frequently diagnosed microsporidian species in humans and a wide range of animals. This study was conducted to assess the prevalence and molecular characteristics of *E. bieneusi* in dairy cattle in Henan Province of central China and the Ningxia Hui Autonomous Region of northwest China.

**Findings:**

Of 879 fresh fecal specimens, 24.3 % (214/879) tested positive for *E. bieneusi* by nested polymerase chain reaction (PCR) based on the internal transcriber spacer (ITS) gene. The highest infection rate, 46.8 % (51/109, *P* < 0.0001), was observed in a group of dairy cattle with diarrhea, located in Ningxia. The age groups with higher infection rates were pre-weaned calves (29.3 %, 127/434, *P* < 0.0001) and post-weaned calves (23.9 %, 63/264, *P* = 0.006). Sequencing and phylogenetic analysis revealed 20 *E. bieneusi* ITS genotypes (15 known and five new), including members of Group 1 and Group 2. Genotypes I and J were detected in 64.5 % (138/214) of the *E. bieneusi* positive specimens.

**Conclusions:**

Genotypes I and J were the dominant genotypes in dairy cattle in the present study. The detection of zoonotic genotypes of *E. bieneusi* in dairy farms indicates that cattle may play an important role as a reservoir host for zoonotic infections.

## Background

The microsporidia are a large and diverse group of obligate intracellular eukaryotic parasites; approximately 1,300 microsporidian species in 160 genera have been reported [1]. *Enterocytozoon bieneusi* is frequently detected in humans, primarily invading the epithelial cells of the small intestine and causing chronic diarrhea and wasting syndrome [2]. *Enterocytozoon bieneusi* has also been frequently reported in livestock, domestic animals and wildlife all over the world [3–5].

More than 200 *E. bieneusi* genotypes have been characterized in humans and animals on the basis of sequence analysis of the ribosomal internal transcribed spacer (ITS) gene [6, 7]. In a phylogenetic analysis, all *E. bieneusi* ITS genotypes were divided into nine groups [7]. The Group 1 is referred to as the human-pathogenic group and the other Group 2 through Group 9, found mostly in specific hosts and wastewater [5, 7, 8]. However, some genotypes (I, J and BEB4) from Group 2 also have recently been reported in humans [4, 9, 10].

Since the first report in three calves in Germany [11], *E. bieneusi* has been commonly detected in cattle, with more than 40 genotypes identified [6, 12]. More importantly, the presence of zoonotic *E. bieneusi* genotypes in bovine milk [13] and the environment [14] indicates the possibility that dairy cattle may play a role in the transmission of *E. bieneusi* to humans or other species. Therefore, it is especially important to identify and genotype bovine *E. bieneusi* isolates, as this is not only a veterinary issue but also a public health concern.

The percentage of zoonotic genotypes of *E. bieneusi* in animals is an important parameter to assess the risk of zoonotic transmission of microsporidiosis in a specific area. The present study was conducted to determine the occurrence and molecular characterization of *E. bieneusi* in cattle in Henan Province of central China and the Ningxia Hui Autonomous Region of northwest China.

## Methods

### Ethics statement

This study was conducted in accordance with the Chinese Laboratory Animal Administration Act (1988) and the study protocol was approved by the Research Ethics Committee of Henan Agricultural University. Permission was obtained from the farm director before the collection of fecal specimens.

### Specimen collection

A total of 879 fresh fecal specimens were collected from Zhengzhou in Henan Province of central China (34°44’N, 113°38’E, mean annual temperature 14 °C, mean annual precipitation 641 mm) and Zhongwei in the Ningxia Hui Autonomous Region of northwest China (37°29’N, 105°41’E, mean annual temperature 11 °C, mean annual precipitation 192 mm). Three farms were sampled: 515, 255 and 109 specimens were collected from Henan farm 1 (collecting time: from June of 2014 to January of 2015, sampled eight times), Henan farm 2 (collecting time: from January to June of 2013, sampled five times) and Ningxia farm (collecting time: October of 2013, sampled once), respectively. The specimens from the Ningxia farm comprised a part of a previous study [15]. Fresh fecal specimens for each animal were collected immediately after defecation on the ground, and stored at 4 ° C before DNA extraction.

### Molecular identification

DNA was extracted with the E.Z.N.A.R.® Stool DNA Kit (Omega Biotek Inc., Norcross, GA, USA) according to the manufacturer’s instructions. For screening *E. bieneusi*, previously described nested PCR assays were used to amplify the internal transcribed spacer (ITS) gene [16]. Amplicons were sequenced on an ABI PRISM™ 3730 XL DNA Analyzer using the BigDye Terminator v3.1 Cycle Sequencing Kit (Applied Biosystems, Foster City, CA, USA). Sequence accuracy was confirmed with bidirectional sequencing, and the program ClustalX 2.0 (http://www.clustal.org/) was used to align the obtained sequences with reference sequences to determine the genotypes. The phylogenetic analysis was conducted with neighbour-joining analysis using the program Mega 5 [7] (http://www.megasoftware.net/). The established nomenclature system was used in naming novel *E. bieneusi* ITS genotypes [7, 8]. Representative nucleotide sequences were deposited in GenBank (Accession numbers: KU245694–KU245706).

### Statistical analysis

The *χ*^2^ test was used to compare the *E. bieneusi* infection rates. Differences were considered significant at P < 0.05.

### Findings and discussion

Among the 879 fecal specimens collected from dairy cattle, 214 (24.3 %, 214/879) were *E. bieneusi* positive. All the three farms showed evidence of *E. bieneusi* infection, with infection rates of 24.1 % (124/515, Henan farm 1), 15.3 % (39/255, Henan farm 2) and 46.8 % (51/109, Ningxia farm), respectively. The highest infection rate, 46.8 % (51/109) in Ningxia, was statistically higher than the other two Henan farms (*χ*^2^ = 41.17, *P* < 0.0001). *E. bieneusi* infection may partially responsible for the diarrhea and death of dairy cattle in Ningxia [15]. The age group with the higher *E. bieneusi* infection rate were the pre-weaned and post-weaned calves with 29.3 % (127/434, *χ*^2^ = 17.66, *P* < 0.0001) and 23.9 % (63/264, *χ*^2^ = 7.68, *P* = 0.006), significantly higher than juvenile and adult dairy cattle (13.3 %, 24/181) (Table 1).

**Table 1:** Genotypes and distribution of *E. bieneusi* in dairy cattle by age group in Henan and Ningxia

Age group (month)		No. of positive / No. of examined (%)	Genotypes (*n*)
Pre-weaned calves (<3)	1 M	76/234 (32.5)	J (30); I (28); BEB4 (3); BEB6 (1); CM8 (10); CHC4 (1); EbpA (1); EbpC (1); O (1)
	2 M	51/200 (25.5)	J (22); I (7); BEB6 (4); CM8 (7); CD6 (1); COS-1 (1); CHC3 (1); CHC5 (1); EbpC (4); D (2); H (1)
	Subtotal	127/434 (29.3)	J (52); I (35); BEB4 (3); BEB6 (5); CD6 (1); CM8 (17); COS-1 (1); CHC3 (1); CHC4 (1); CHC5 (1); EbpA (1); EbpC (5); D (2); H (1); O (1)
Post-weaned calves (3–12)	3 M	16/85 (18.8)	J (4); I (2); BEB4 (1); BEB6 (4); CM8 (1); CHC2 (1); COS-1 (2); EbpA (1)
	4 M	15/59 (25.4)	J (8); I (2); BEB4 (2); CHC1 (1); CHG2 (1); COS-1 (1)
	5 M	15/46 (32.6)	J (8); I (3); BEB4 (3); COS-1 (1)
	6 M	6/35 (17.1)	J (1); I (1); BEB4 (3); CHG3 (1)
	>6 M	11/39 (28.2)	I (10); BEB4 (1)
	Subtotal	63/264 (23.9)	J (21); I (18); BEB4 (10); BEB6 (4); CM8 (1); CHC1 (1); CHC2 (1); CHG2 (1); CHG3 (1); COS-1 (4); EbpA (1)
Juveniles & Adults (>12)	24/181 (13.3 %)		J (4); I (8); BEB4 (2); BEB6 (8); BEB8 (1); EbpC (1)
Total	214/879 (24.3 %)		J (77); I (61); BEB4 (15); BEB6 (17); BEB8 (1); CD6 (1); CM8 (18); COS-1 (5); CHC1 (1);CHC2 (1); CHC3 (1); CHC4 (1); CHC5 (1); CHG2 (1); CHG3 (1); EbpA (2); EbpC (6); D (2); H (1); O (1)

Abbreviation: *M*, month

A total of 20 *E. bieneusi* ITS genotypes were obtained from 214 successfully sequenced specimens from dairy cattle. Among them, 15 were known genotypes (including genotype I, J, BEB4, BEB6, BEB8, CD6, CHG1, CHG2, CM8, COS-1, EbpA, EbpC, D, H and O), and five were new genotypes (named CHC1–CHC5).

Genotypes I and J were found in all three farms, which were previously known to be present in cattle, sheep, goats, cats, yaks, pigeons and captive wildlife [6, 12, 17]. Genotype J was found in 77 (36.0 %, 77/214) of the *E. bieneusi-*positive specimens, including 44 (35.5 %, 44/124), 8 (20.5 %, 8/39) and 25 (49.0 %, 25/51) of the specimens from Henan farm 1, Henan farm 2 and Ningxia farm, respectively. Genotype I was found in 61 (28.5 %, 61/214) of the detected *E. bieneusi* specimens, including 47 (37.9 %, 47/124), 7 (17.9 %, 7/39) and 7 (13.7 %, 7/51) specimens from Henan farm 1, Henan farm 2 and Ningxia farm, respectively. Genotypes I and J were found in 64.5 % (138/214) of the detected *E. bieneusi* positive specimens, and these were the dominant genotypes in present study. Also, similar results have been reported by Jiang et al*.* (41.9 %, 13/31) [6] and Ma et al*.* (71.4 %, 25/35) [18] in China. On the contrary, genotype O (65 %, 26/40) was the dominant genotype detected by Zhao et al*.* in Heilongjiang Province, China [19], and the genotypes I and J (10 %, 4/40) were the second and third dominant genotypes. Genotypes I and J were also the dominant genotypes found in Argentina (6/10) [12], Czech Republic (6/6) [20], China (17/35) [4], Germany (7/10) [11], and two studies in the United States (17/17; 167/285) [21, 22].

Genotype D is considered to exhibit the widest host range and has been detected in more than 25 species of mammals [5, 8, 23]; this was also the most common genotype infecting humans [23], and was identified in two specimens in this study. The zoonotic genotypes EbpC and BEB4, previously identified in cattle, pigs and humans, and the genotypes BEB6 and BEB8, previously identified in cattle, also were found in this study. The seven genotypes CD6, CHG2, CHG3, CM8, COS-1, EbpA and H, which were previously identified in dogs, sheep, goats, pigs and monkeys [6, 12], were first identified in dairy cattle.

**Fig 1:**
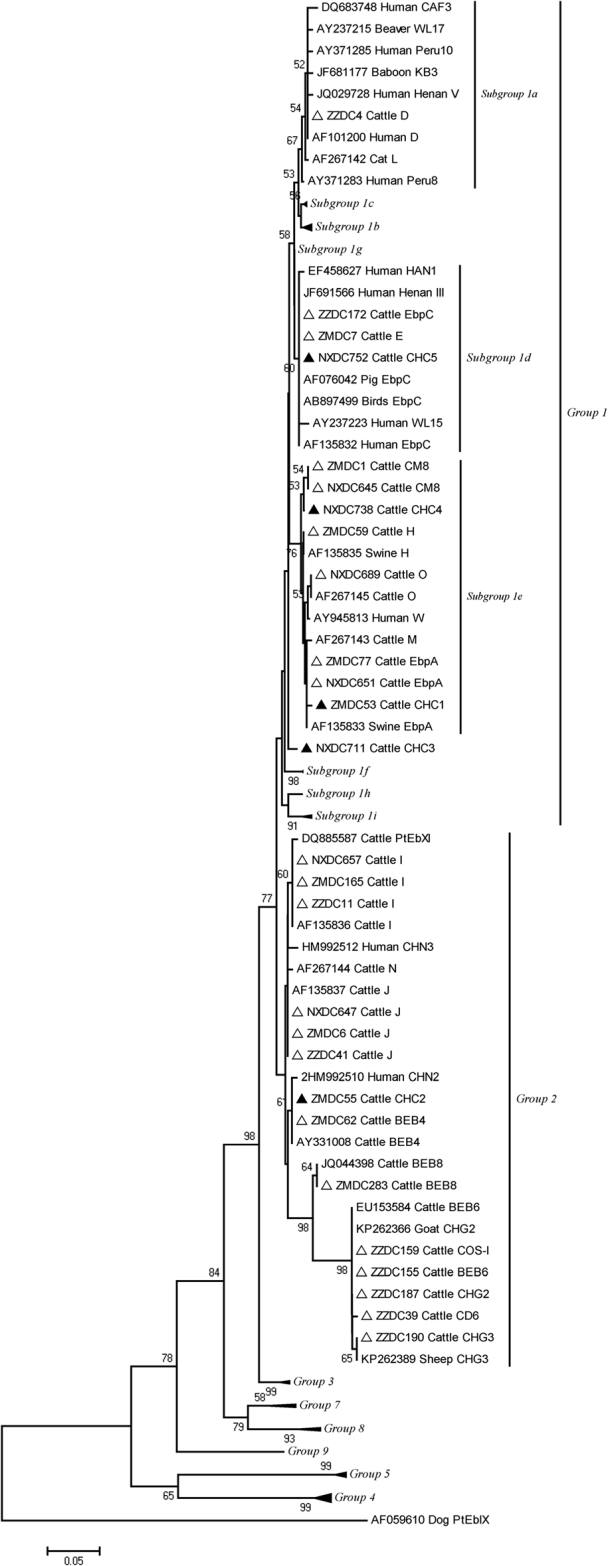
Phylogenetic relationships of the *E. bieneusi* genotypes identified in this study and other reported genotypes. The phylogeny was inferred with a neighbour-joining analysis of the ITS sequences based on distances calculated with the Kimura two-parameter model. Bootstrap values > 50 % from 1,000 replicates are shown on the nodes. The genotypes detected in this study are shown with triangles; known genotypes observed in this study are marked with open triangles and novel genotypes are indicated by filled triangles

The sequence diversity was observed in the identified *E. bieneusi* ITS genotypes. Among the 20 *E. bieneusi* ITS genotypes detected, six known genotypes (D, EbpA, EbpC, CM8, H and O) and four novel genotypes (CHC1, CHC3, CHC4 and CHC5) fell into the category previously described as a zoonotic Group 1 in a phylogenetic analysis. In contrast, nine known genotypes (I, J, BEB4, BEB6, BEB8, COS-1, CD6, CHG2 and CHG3) and one novel genotype (CHC2) were categorized as Group 2 (Fig. 1). The five novel genotypes (CHC1–CHC5) obtained in this study are genetically closely related to human-pathogenic genotypes, with one to three base substitutions. Four of these novel genotypes were placed into Group 1; the other, CHC2, was characterized as Group 2 (Fig. 1). In this study, 76.6 % (161/214) of the identified genotypes were potentially zoonotic pathogens. Therefore, cattle may be a reservoir for zoonotic *E. bieneusi* genotypes, and these infections may be not only a veterinary issue but also a public health threat.

## Conclusions

This study is the first report of the genotypes CD6, CHG2, CHG3, CM8, COS-1, EbpA and H, and five novel *E. bieneusi* ITS genotypes (CHC1–CHC5) in dairy cattle. Genotypes I and J were the dominant genotypes in dairy cattle in present study. The detection of zoonotic genotypes of *E. bieneusi* in dairy farms indicates that cattle may play an important role in the epidemiology of *E. bieneusi* as a reservoir host for zoonotic infections.
